# Class II subdivision correction with clear aligners using intermaxilary elastics

**DOI:** 10.1186/s40510-018-0221-5

**Published:** 2018-09-01

**Authors:** Luca Lombardo, Anna Colonna, Antonella Carlucci, Teresa Oliverio, Giuseppe Siciliani

**Affiliations:** Postgraduate School of Orthodontics, University of Ferrara, Ferrara, Italy

## Abstract

**Background:**

To describe an esthetic orthodontic treatment using aligners in an adult patient with class II subdivision associated with crowding and dental crossbite. An 18-year-old hyperdivergent male patient with skeletal class II from mandibular retrusion presented for an orthodontic treatment. Occlusally, the patient presents class II subdivision, crossbite at tooth 4.4, an upper midline deviated towards the left with respect to the lower and facial midlines, and slight crowding in both arches. The patient refused conventional fixed multibracket treatment in favor of aligners. Pre- and post-treatment records as well as 1-year follow-up records are presented.

**Findings:**

Treatment objectives were achieved in 12 months, and the patient was satisfied with the functional and esthetic outcomes, which were stable at 1 year.

**Conclusion:**

Combining aligners with appropriate auxiliaries is an efficacious means of resolving orthodontic issues such as class II, dental crossbite, and crowding in a time-frame comparable to that of conventional fixed orthodontics. Furthermore, this system is associated with optimal oral hygiene and excellent esthetics.

## Background

Nowadays, there is a growing demand for esthetic treatment among both adolescents [1] and adults [2]. Indeed, a recent study estimated that 45% of adults are unhappy with their smile and that 20% of these have considered undergoing orthodontic treatment to improve their appearance [3].

Hence, aligner systems must now be able to treat various types of malocclusion, and over recent years, many studies have shown their great efficacy in correcting crowding, misalignment and diastems, and even complex cases featuring extraction, open-bite, and poor occlusal relationships [4–8].

## Case report

This case report describes an adult male patient with class II subdivision malocclusion, dental crossbite, and crowding treated successfully with aligners.

### Diagnosis and etiology

**Fig. 1:**
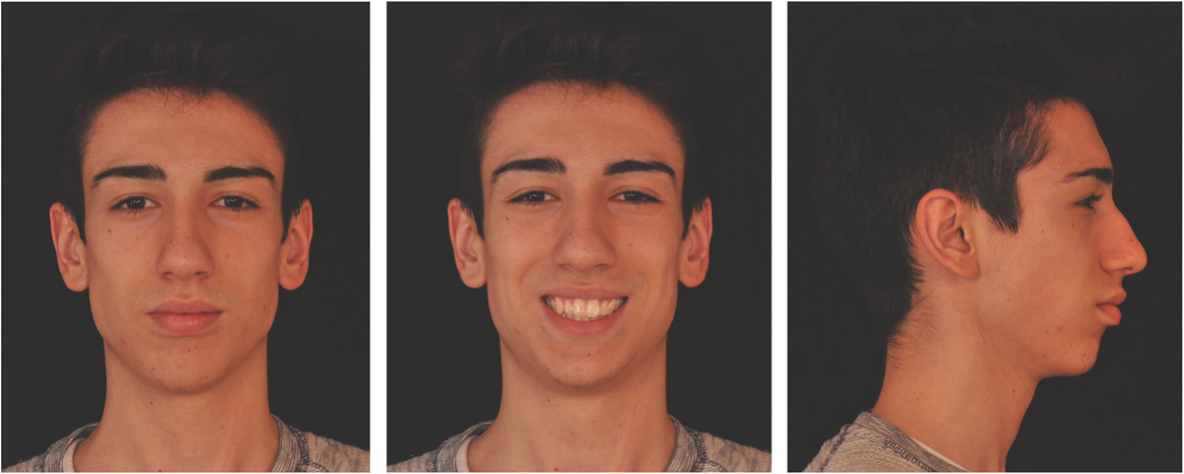
Pre-treatment extraoral photos

An 18-year-old hyperdivergent male patient presented for treatment. Extraoral photos (Fig. 1) and frontal examination revealed good incisor exposure; however, buccal corridors and upper midline deviation towards the left with respect to the facial midline were present. The profile had a convex aspect characterized by mandibular retrusion and increased lower facial height.

Clinical examination revealed class II subdivision with lower midline deviation towards the right of the upper midline, dental crossbite, slight crowding in both arches, and small alteration of the upper right lateral incisor morphology.

**Fig. 2:**
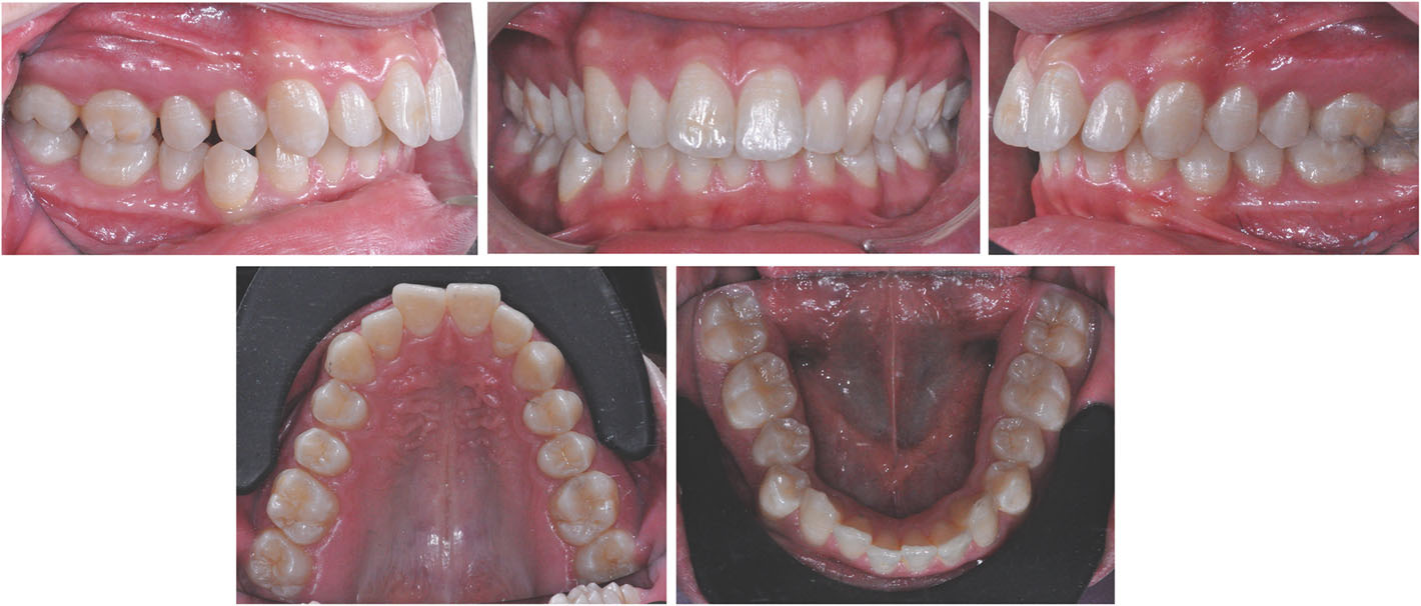
Pre-treatment intraoral photos

Periodontal biotype and oral hygiene were good (Fig. 2).

**Fig. 3:**
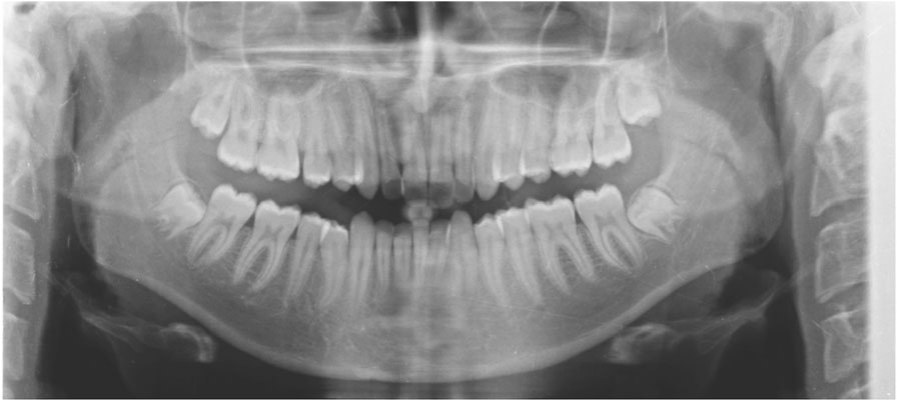
Pre-treatment panoramic radiograph

Panoramic radiography revealed full dentition, a lack of bone defects, no infection and no temporomandibular joint abnormalities (Fig. 3).

**Fig. 4:**
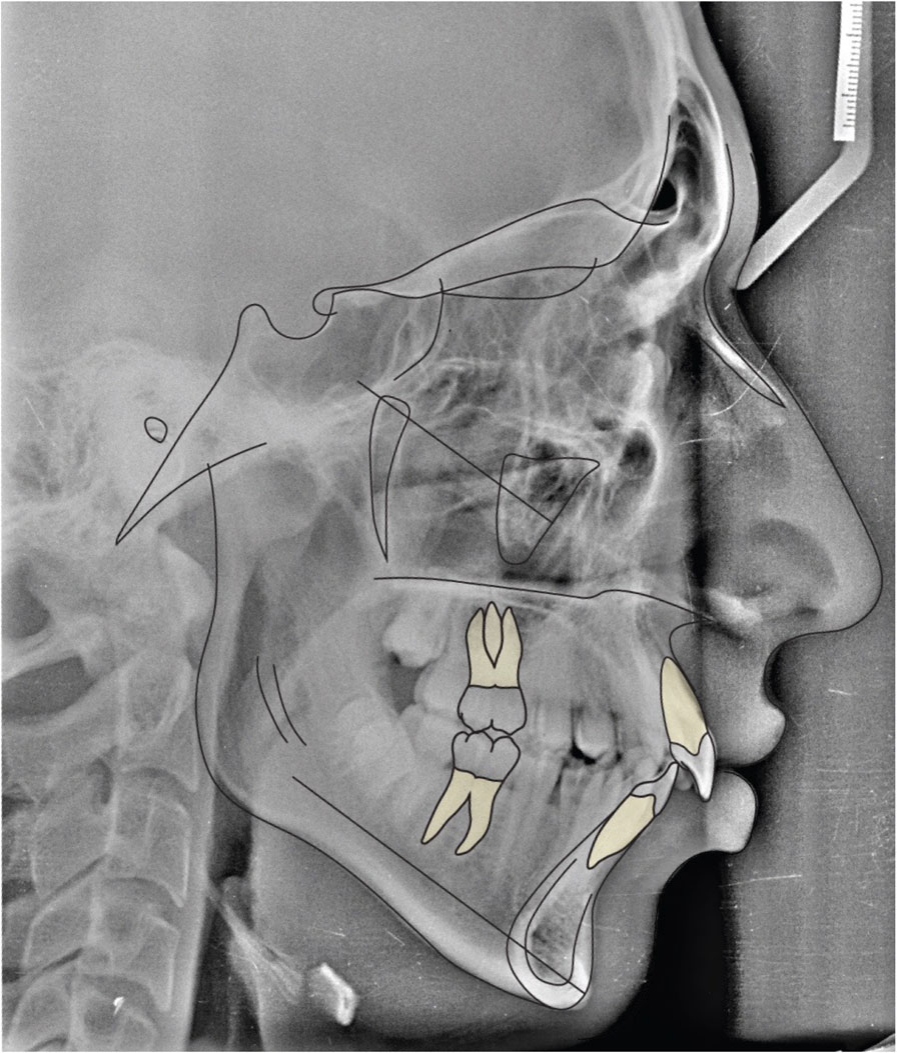
Initial radiographs and cephalometric tracing

Latero-lateral teleradiograpy (Fig. 4) showed skeletal class II from mandibular retrusion, and a hyperdivergent facial type; maxillary incisor were proclined and mandibular incisors had a correct inclination. Overbite and overjet were increased as reported in Table 1.

**Table 1:** Pre- and post-treatment cephalometric values

	Pre-treatment value	Post-treatment value	Ref. value	Standard deviation
SNA (°)	79.0	81	82.0	3.5
SNB (°)	72.0	74.8	80.0	3.0
ANB (°)	7.0	6.2	2.0	2.0
Wits appraisal (mm)	4.7	4.0	0.0	1.0
FMA (°)	31.5	30.3	26.0	5.0
MP-SN (°)	40.5	40.3	33.0	6.0
Palatal-mand-angle (°)	27.0	28.0	28.0	6.0
PP-OP (°)	5.3	5.6	10.0	4.0
Mand plane to occ plane (°)	21.5	22.0	18.0	5.0
U1-APo (mm)	9.8	6.8	6.0	2.2
L1-APo (mm)	3.7	3.6	2.0	2.3
U1-palatal plane (°)	118.0	111.0	110.0	5.0
IMPA (°)	92.5	96.5	95.0	7.0
Overjet (mm)	5.5	2.6	3.5	2.5
Overbite (mm)	4.5	2.5	2.5	2.0

Clinical examination showed no sign of bad habits.

### Treatment objectives

**Fig. 5:**
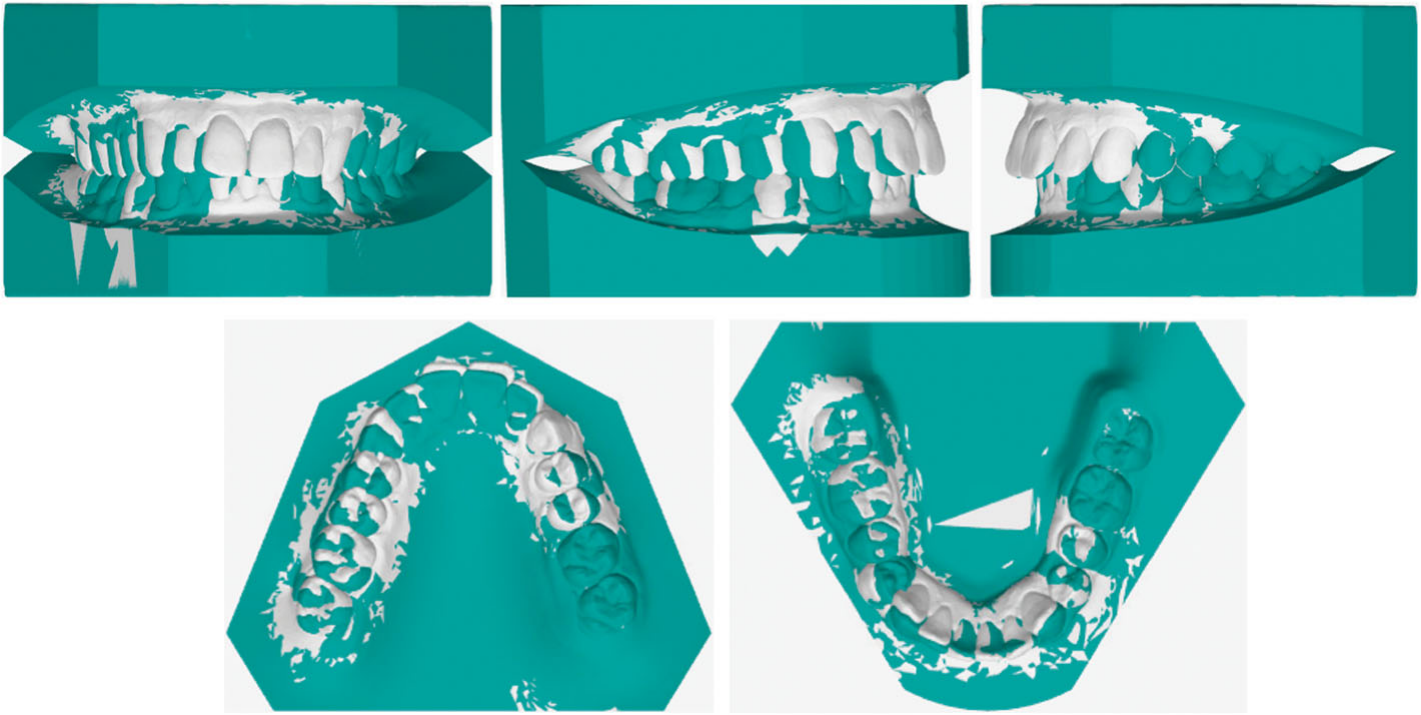
Set-up viewer: the initial occlusion is shown in white and the post-treatment objectives in green

The primary objective was to achieve a molar and canine class I and centering the upper midline with the lower and facial midlines. Additional objectives were to correct the crowding and dental crossbite, obtain ideal overjet and overbite (Fig. 5), improve facial esthetics, and reduce black buccal corridors during smile.

### Treatment alternatives

As there were no major skeletal discrepancies, a combined orthodontic/surgical approach was ruled out. Fixed multibracket treatment with extraction of four premolars was considered, but also excluded due to potential worsening of the profile. The patient was therefore offered a treatment plan involving unilateral distalization by fixed multibracket appliances in order to center the upper midline with the lower and facial midlines. However, the patient refused this option due to the unsightliness of the device, and we therefore agreed upon a non-extractive treatment with F22 aligners (Sweden & Martina, Due Carrare, Italy) for unilateral distalization and mesialisation of the lower arch in order to correct the class II relationship.

### Treatment progress

The virtual set-up dictated 20 treatment steps for each arch. To achieve upper midline correction, the plan involved distorotation of teeth 1.6 (22°) and 1.7 (13°) in association with distalization. The use of class II elastics had a double function: the anchorage, used to obtain simultaneous distalization of the elements of the quadrant I and support the correction of the lower midline.

In the lower arch, the plan involved mesorotation of teeth 4.6 and 4.7 associated with mesial tipping. The plan also involved alignment of the arches and retroclination of the upper incisors.

In order to achieve correct alignment and valid intercuspidation, vestibular grip points on teeth 1.6, 1.7, 4.5, 4.4, and 4.3 were planned, alongside 0.2 mm of stripping at each interproximal point in the lower right sector, from the mesial surface of tooth 4.6 to the distal surface of 4.2.

**Fig. 6:**
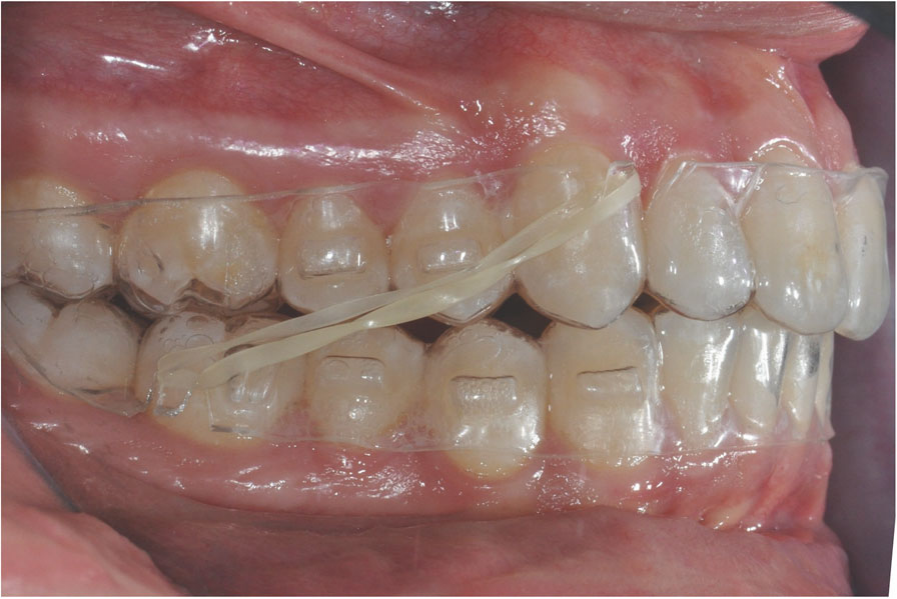
Combine use of aligners and class II intermaxillary elastics

In order to promote achievement of class I, 6 oz. intermaxillary elastics were to be hooked directly on the apposite notches in the aligners at the upper canines and lower first molars from step 1 onwards (Fig. 6). The patient was instructed to wear each aligner for 22 h per day and to move on to the next one in the series after 14 days.

After 10 months of treatment, the treatment objectives had been successfully fulfilled, although it was necessary to plan another three steps per arch for detailed finishing of the case and complete class correction. Specifically, the derotation of teeth 4.5, 1.6, and 1.5 was improved.

### Treatment results

**Fig. 7:**
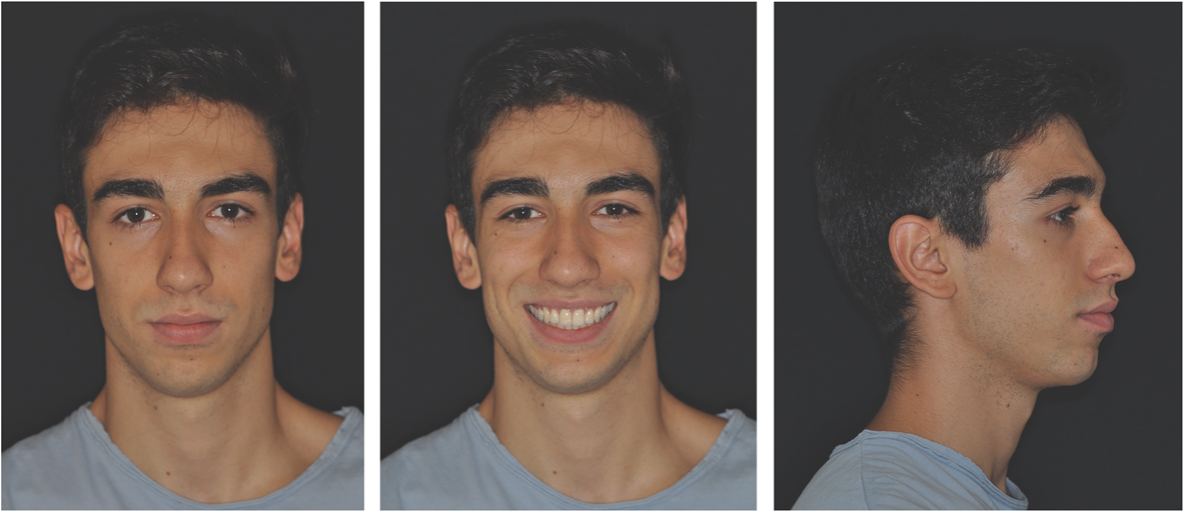
Post-treatment extraoral photos

**Fig. 8:**
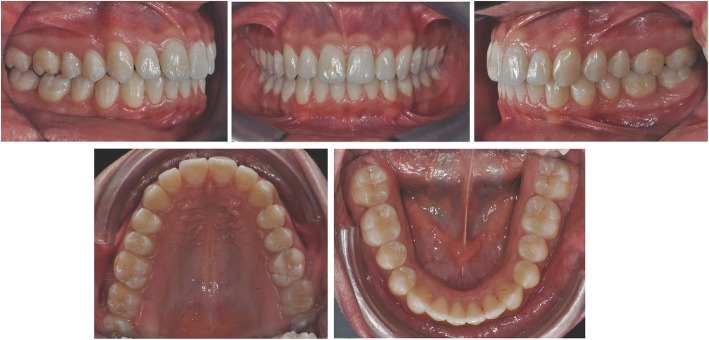
Post-treatment intraoral photos

**Fig. 9:**
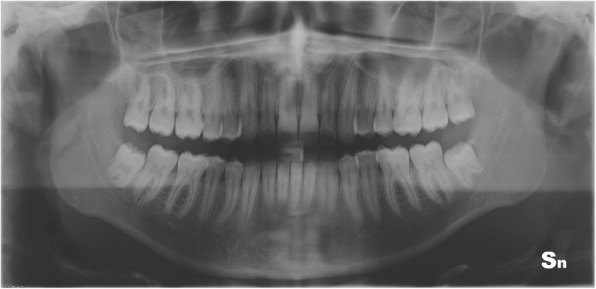
Post-treatment panoramic radiograph

**Fig. 10:**
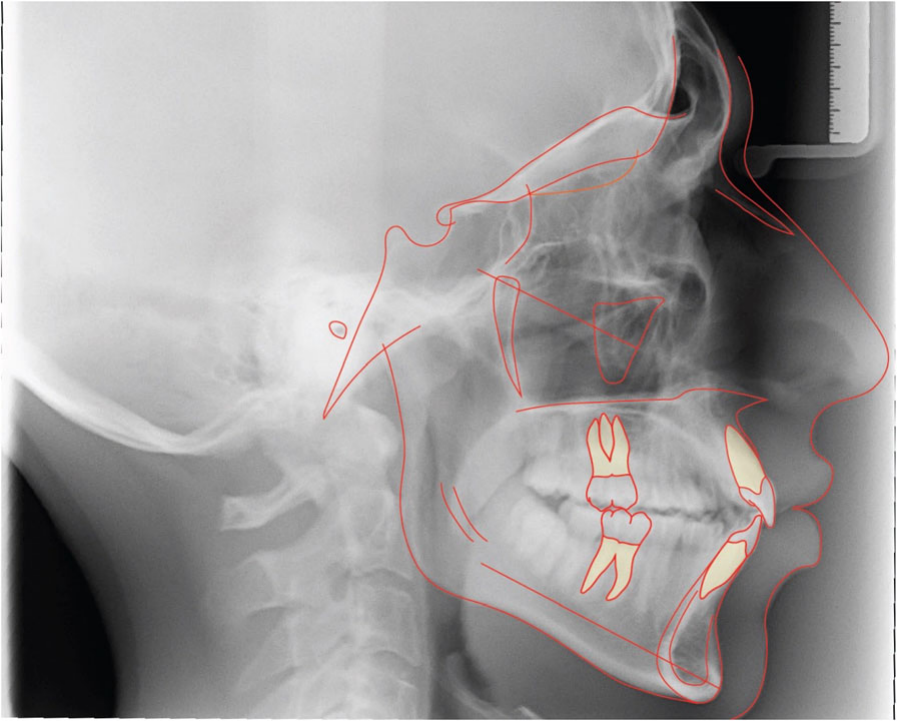
Final radiographs and cephalometric tracing

**Fig. 11:**
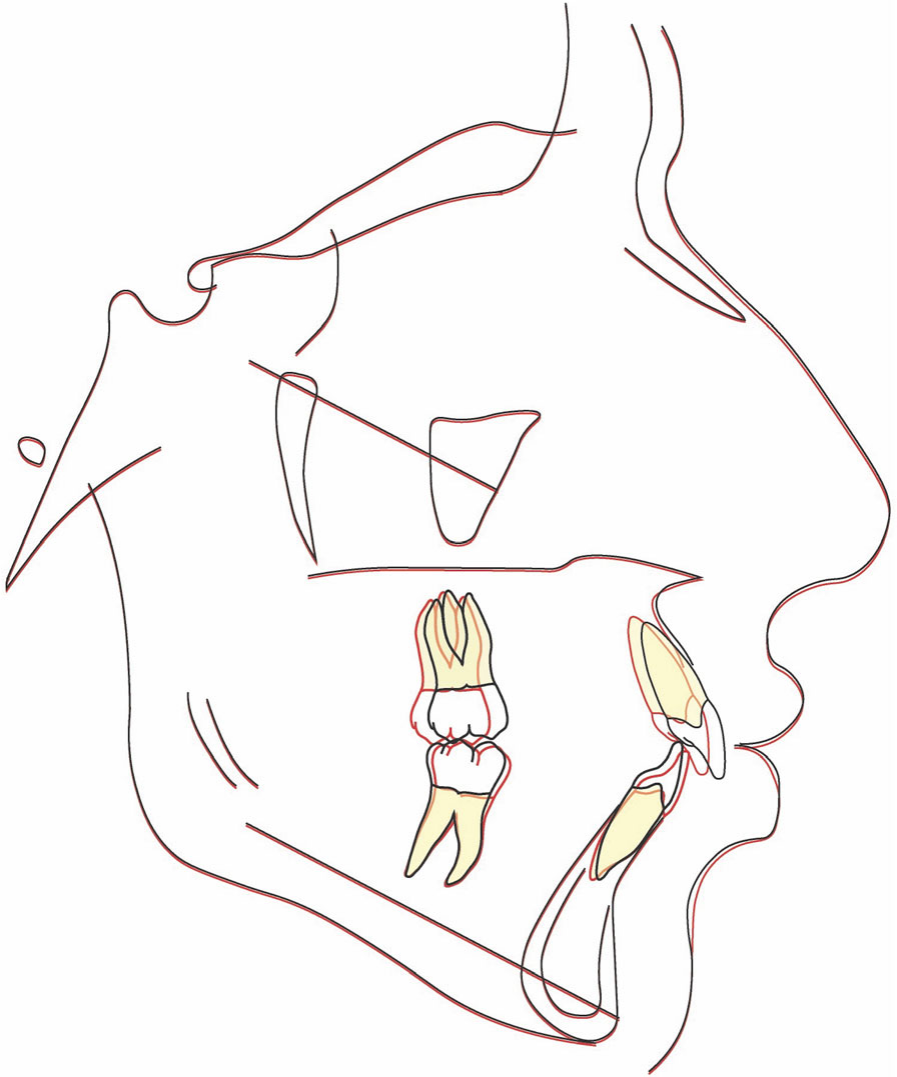
Superimposition on the anterior cranial base. Made by “The structural Method” developed by Professor Arne Björk. The stable anatomical structures of anterior cranial base are: • The inner contour of the anterior wall of Sella Turcica. • The mean intersection point of the lower contours of the anterior clinoid processes and the contour of the anterior wall of saddle, Walkers’s point. • The anterior contours of the middle cranial fossae. The contours of the bilateral fronto-ethmoidal crests. • The cerebral surfaces of the orbital roofs

**Fig. 12:**
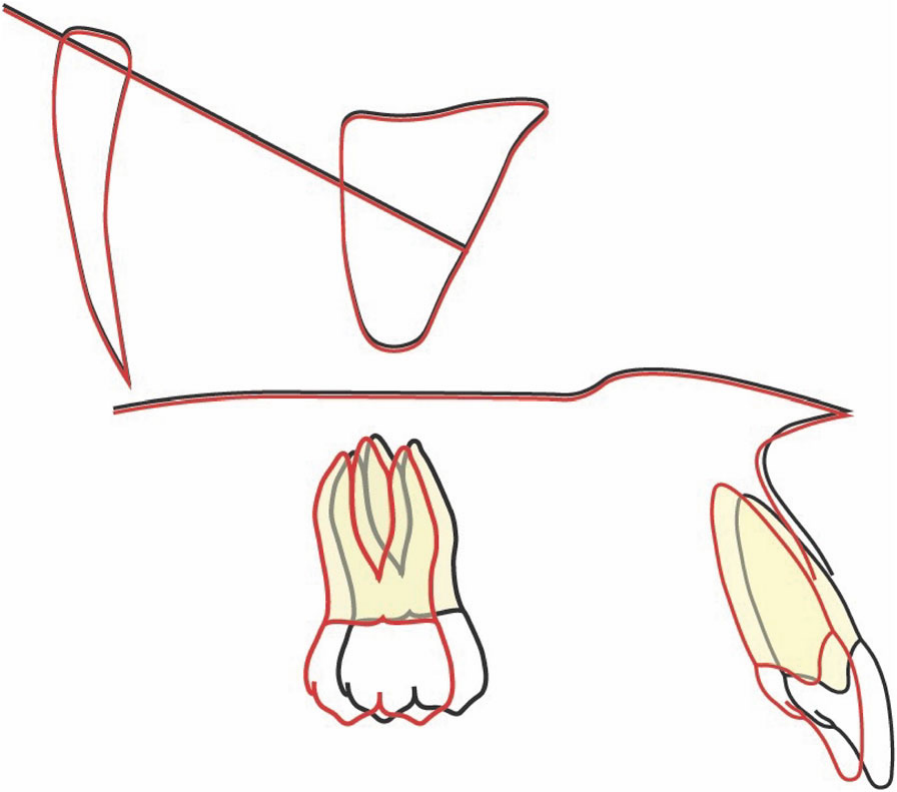
Superimposition of the maxilla. The only stable structure in the maxilla is the anterior contour of the zygomatic process [9]

**Fig. 13:**
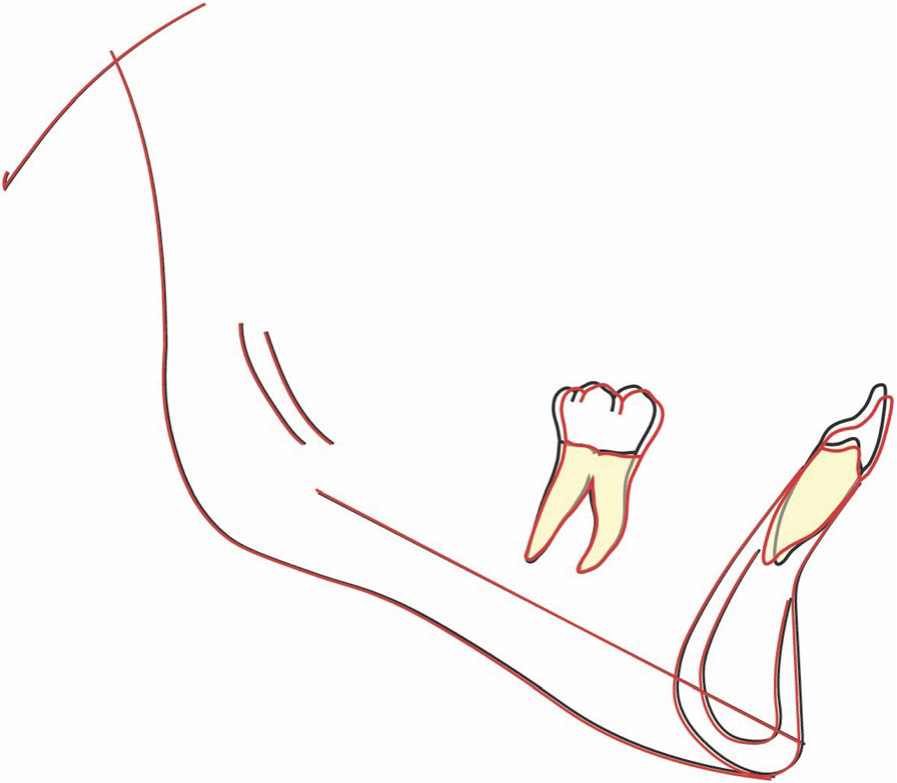
Superimposition of the mandible. The stable anatomical structures of the mandible are: • The anterior contour of the chin.• The inner cortical structure at the inferior border of the symphysis. • Trabecular structures related to the mandibular canal [10]

Post-treatment records demonstrate satisfactory final results with all objectives achieved. Extraoral photos show a good profile, correct incisor exposure during smile and the absence of buccal corridors (Fig. 7). Intraoral examination reveals the achievement of all planned objectives, namely class I, centered midlines, and crowding correction (Fig. 8). Post-treatment panoramic radiography (Fig. 9) showed good root parallelism, no sign of crestal bone height reduction, and no evidence of apical root resorption. Cephalometric indices and post-treatment latero-lateral teleradiograpy show good vertical control and proclination of the lower incisors (Table 1). Superimposition of pre- and post-treatment cephalometric tracings (Figs. 4, 10, 11, 12, and 13), carried out according to the methodology described in the images captions as developed by Professor Arne Björk [9, 10], highlight the distal tipping movement of the right upper sector, the retroclination achieved at the upper incisors, and the proclination of the upper incisors with respect to the basal bone—an acceptable outcome due to the morphology and conformation of the patient’s symphysis.

**Fig. 14:**
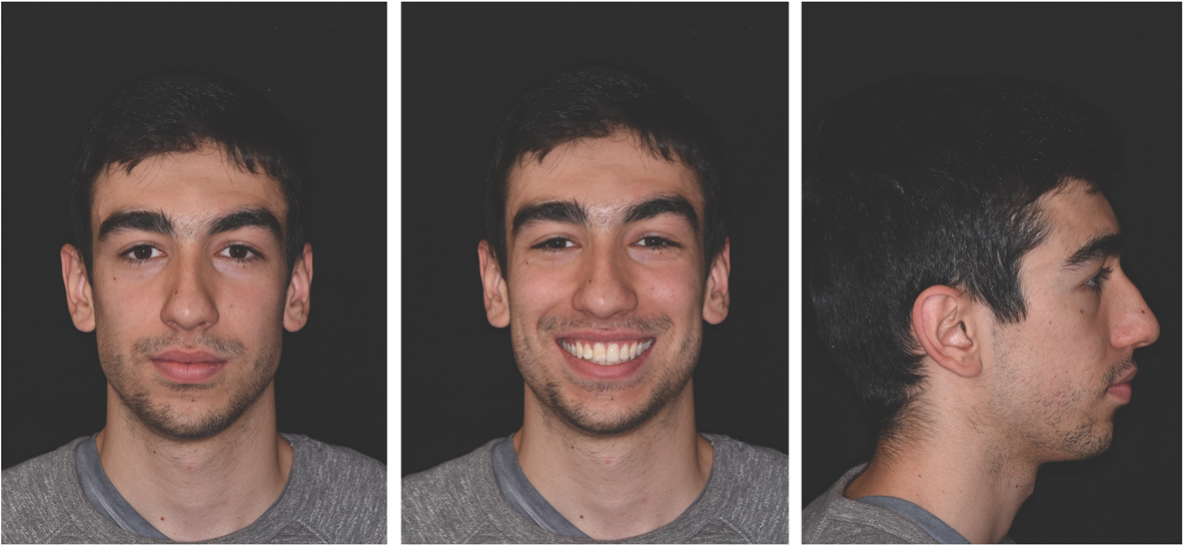
Extraoral photos at 1 year

**Fig. 15:**
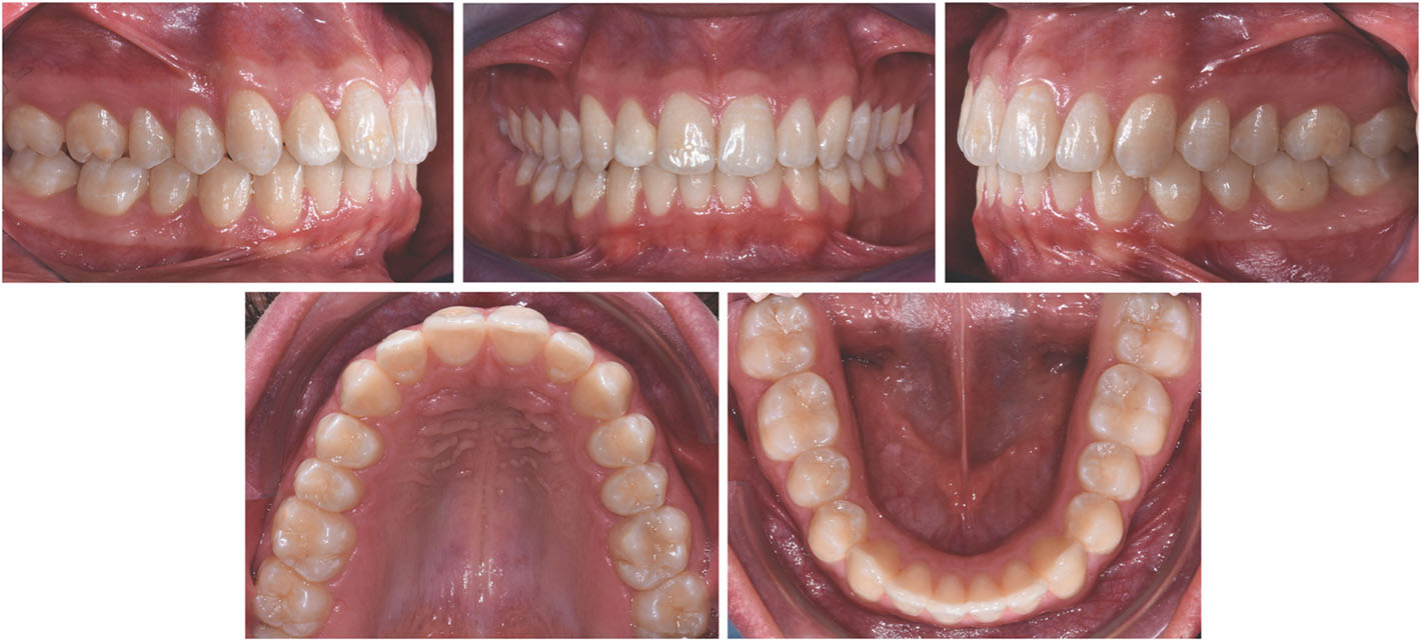
Intraoral photos at 1 year

Check-up at 1 year demonstrates the excellent stability of results (Figs. 14 and 15).

The last pair of aligners was used for retention due to the elastic propriety of the thermoplastic material [11].

Restoration of the 1.2 was performed in order to improve its morphology.

## Discussion

Aligners associated with intermaxillary elastics enabled resolution of the malocclusion within a treatment time comparable with that required for conventional fixed orthodontics, providing the patient with a comfortable, practical, and esthetic appliance. This case report is very similar to those presented in 2010 by Schupp et al. [12]. In our case, in order to prevent unwanted extrusion and/or rotation and further enhance the esthetics, we used notches in the aligners at the upper canines and lower first molars rather than buttons bonded directly onto the teeth for attaching the intermaxillary elastics. Unfortunately, a direct comparison of the cephalometric indices, especially pertaining to the lower incisor proclination, was not possible, as these were not provided in the report.

From a biomechanical perspective, the dental movements occurred as planned, thanks to the fact that they were planned within the correct range of predictability [13] and the excellent properties of the material that the F22 aligners are made of [11].

In accordance with findings from Janson et al. [14], and due to the age of the patient, the movements brought about by the use of intermaxillary elastics were predominantly dentoalveolar in nature and led to a slight reduction in the SNA angle, a slight increase in the IMPA, and retroclination of the upper incisal sector. Their main effect was to provide anchorage for the upper arch, thereby promoting distal tipping and retroclination of the upper incisors.

The treatment plan selected proved to be a winning solution not only in terms of biomechanics, but also as regards esthetics and periodontal health. Indeed, previous studies [15] have shown that fixed multibracket appliances, whether labial or lingual, are associated with an increase in plaque retention, which in turn may cause an increase in *S. mutans* concentration and gingival inflammation. Furthermore, the use of such devices can increase the chromium and nickel concentration in a patient’s mucosa, potentially resulting in damage to DNA [16], whereas aligners have not been linked to any type of cytotoxicity [17].

Moreover, Abbate et al. [18] revealed the microbiological and periodontal changes that may occur during orthodontic treatment; comparing aligners and fixed appliances, they found that aligners were associated with greater compliance, better oral hygiene, less accumulation of plaque, and less gingival inflammation than fixed appliances. These findings are in line with those reported in a previous study by Mietheke et al. [19], and in another that showed an increase in periodontopathic bacteria associated with a worsening of periodontal health in fixed multibracket orthodontics with respect to aligners [20].

## Summary and conclusions

Combined use of aligners and auxiliaries is an efficacious means of resolving orthodontic issues such as class II, dental cross-bite, and crowding within a time-frame comparable to conventional fixed orthodontics, but with excellent esthetics and oral hygiene.
